# Validation of *Cyber Test* for *Future Soldiers*: A Test Battery for the Selection of Cyber Soldiers

**DOI:** 10.3389/fpsyg.2022.868311

**Published:** 2022-04-14

**Authors:** Patrik Lif, Teodor Sommestad, Pär-Anders Albinsson, Christian Valassi, Daniel Eidenskog

**Affiliations:** The Division of C4ISR – Command, Control, Communications, Computers, Intelligence, Surveillance and Reconnaissance, Swedish Defence Research Agency, Linköping, Sweden

**Keywords:** cyber test, cyber soldiers, CTFS, validity, cognitive ability, computer knowledge

## Abstract

To facilitate recruitment of conscript cyber soldiers in Sweden, the *Cyber Test* for *Future Soldiers* (*CTFS*) was developed as a complement to the existing generic enrolment test (*I-prov 2000*). Consisting of several parts, *CTFS* measures different aspects of computer-related knowledge and cognitive abilities that we believe are of particular relevance to cyber security. This article describes the evaluation regarding *CTFS’s* validity and reliability based on data from 62 conscripts that took the test and the 27 selected conscripts finishing the training 1 year later (being the first to do so). Reliability was predicted through internal consistency and test–retest measures. Cronbach’s alpha for *CTFS* ranges from 0.79 to 0.86 whereas the test–retest reliability is 0.60. Evidence regarding validity was collected based on test content and internal structure. Convergent, discriminant, and predictive evidence are also presented. Perhaps most importantly, *CTFS* could predict the cyber soldiers’ performance during their training and added incremental validity to *I-prov 2000*. When adjusted for range restriction, *I-prov 2000* predicted 20% of the variation in course score, while 34% of the variance was explained when *CTFS* was used in conjunction with *I-prov 2000*. The results show that *CTFS* already in its first version is adding value for the Swedish Armed Forces.

## Introduction

A multitude of organizations need to combat a growing cyber threat. Meeting this need requires qualified personnel. However, recruiting and hiring qualified cyber security personnel is a challenge in the current job market. As described in previous research (Allen and Herr, 2019; Markow et al., 2019; ISC2, 2020; Kaminska and Silomon, 2020), there is a general shortage of qualified personnel. In the military, there are generally stricter requirements on cyber security personnel than in the civil domain. For instance, recruits to the military need to hold certain values, pass both integrity evaluations and security clearances and often also meet certain physical conditions, which in turn limits the number of potential recruits. The military has a tradition of using standardized tests to assess candidates’ suitability for different roles. This tradition has prompted initiatives aiming to identify people with an aptitude for cyber security among existing personnel or military recruits, to enable efficient recruitment to internal education and training programs. For instance, the US Air Force has developed a test called the *Cyber Test* (Trippe et al., 2014; Koch et al., 2018), IBM has developed the *Defence Cyber Aptitude Test* for the British Ministry of Defence (IBM, 2018), and SANS (SANS™ Institute, 2021) has developed a test called *Cyber Talent Enhanced*.

This paper describes a test called *Cyber Test* for *Future Soldiers* (*CTFS*) as well as an initial validation. The test was developed in order to select conscripts for basic education as cyber soldiers in the Swedish Armed Forces. The test aims to complement existing tests and measure attributes believed to be of particular importance for individuals serving as cyber soldiers. The *CTFS* validation process extends over a period of approximately 5 years and includes the three phases selection, training, and service. The initial validation of *CTFS* presented in this paper was performed on the first cohort selected with *CTFS*. This cohort has completed their training.

The remainder of this paper is structured as follows: Section “Related Works” describes related works, primarily other research on test batteries for measuring cyber security aptitude. Section “Cyber Test Future Soldiers” describes the components of the test battery and their rationale. Section “Materials and Methods” describes the validation procedure. Section “Results” describes the results of the validation. Section “Discussion” is a discussion on methodological issues, test validity, and future work.

## Related Work

The identification of individuals with a particular attitude, talent, or potential with regard to cyber security is closely related to the problem of predicting individuals’ future performance in roles related to cyber security. Outside the cyber security domain, a considerable body of research is available on predictors of job performance and the validity of different criteria for selecting suitable recruits. The general idea is to infer how well one important variable (e.g., superior ratings) is predicted by other variables (e.g., intelligence). The validity of a prediction variable is typically reported as the correlation coefficient (abbreviated “r”) and the validity of a set of variables is typically reported as the coefficient of multiple correlation (abbreviated “R”).

Schmidt and Hunter (1998) provided an overview of the validity of different variables in personnel selection. They reported that the best predictors of job performance ratings are work sample tests (*r* = 0.54), measures of cognitive ability (*r* = 0.51), and structured interviews (*r* = 0.51). Schmidt and Hunter (1998) also found two combinations that stand out as the most valid and practical ones in personnel selection: cognitive ability combined with integrity tests (*R* = 0.65), and cognitive ability combined with structured interviews (*R* = 0.63).

Thus, there are good reasons for organizations to use tests to screen potential recruits. Accordingly, a number of tests have been developed specifically to measure candidates’ suitability for cyber security work.

The *Cyber Test* was developed by the US Air Force in 2008 as a cognitive subtest of the US Armed Services *Vocational Aptitude Battery* (*ASVAB*). The test was originally referred to as the *Information Communications Technology Literacy* test. When evaluated by Trippe et al. (2014), the test consisted of 206 items: 148 items measuring knowledge, 44 items related to logic, and 14 items related to biodata (e.g., past performance). The evaluation was performed on military students. The students test grades were better predicted by the *Cyber Test* (*r* = 0.64) than by general biodata (*r* = 0.37), the *Electronics Information Test* (*r* = 0.53) and *Figural Reasoning* (*r* = 0.45). However, the *Cyber Test* was not as good as the generic *Armed Forces Qualification Test* (*r* = 0.73). When the *Cyber Test* was added to the *Armed Forces Qualification Test*, an additional predictive validity of 0.05 was obtained. When used to predict students passing or failing a course it also added additional predictive power to the more general *Armed Forces Qualification Test*. It has since been further developed for different military roles and specialties, and validation is ongoing (Trippe and Koch, 2019).

The *Defence Cyber Aptitude Test* (*DCAT*) was developed in collaboration between the International Business Machines Corporation (IBM) and the British Ministry of Defence (Keeley et al., 2017; IBM, 2018). The purpose of the test is to find soldiers in the British Armed Forces with abilities relevant to the cyber area and provide them with specific education and training to become cyber soldiers. The test contains several parts measuring eight dimensions of behavior/personality (e.g., conformity) and five cognitive abilities (e.g., error identification). Limited information is available about the test, especially concerning its validity.

Another initiative related to the selection of cyber security personnel is the *Cyber Aptitude and Talent Assessment* (*CATA*) framework (Campbell et al., 2015). In contrast to a general intelligence-based model, this framework proposes a cybersecurity model with two major components: critical thinking and a job-specific component. Critical thinking includes measures of working memory and reasoning. The job-specific component includes measures of constructs that match the demands of a particular job, which can include cognitive abilities or non-cognitive attributes. Moreover, schematics show that different cyber jobs can be described according to the two dimensions of proactive versus reactive and real-time versus deliberate (Campbell et al., 2015). Some data that partially support this notion are presented in Campbell and Bradley (2018). It measures 15 traits categorized as critical thinking (e.g., visuospatial working memory, rule induction, and complex problem-solving), deliberate action (ability to delay closure and the ability to weight risk and reward), real-time action (psychomotor speed, perceptual speed, and resistance to interfering information), proactive thinking (integrate information into an accurate mental model), and reactive thinking (anomaly detection and vigilance; Coovert et al., 2019). According to Coovert et al. (2019), some validation work has been carried out on *CATA*, but further reviews are required.

Coovert et al. (2019) identified aptitudes and traits required for success in selecting recruits and officers in the United States Air Force using job analysis data from archival information and ratings of subject matter experts. The traits and aptitudes found to be related to performance were analytical thinking, adaptability, dependability, persistence, situational awareness, active learning, decision making, deductive reasoning, and systems thinking. A computer simulation was developed to measure some of these.

The *Cyber Test*, *DCAT*, *CATA*, and the tool developed by Coovert et al. (2019) were all developed to predict future success in roles related to cyber security. However, their contents differ. They all include measures of cognitive ability although with different selections. Common ones include spatial ability, error identification, systems thinking, and analytical or critical ability. Two of the tests measure personality traits while only the *Cyber Test* measures current cyber security knowledge. Furthermore, only the *Cyber Test* has been publicly reported to predict performance on cyber-related tasks (through course scores).

In addition to the above-mentioned tests, there is research not operationalized in tests that assess predictors of performance on cyber-related tasks. Dreibelbis (2019) proposed that various measures of cognitive ability, personality, and motivation would predict performance on tasks related to cyber security, and tested these in relation to superiors’ ratings of 139 persons working with cyber security in various capacities. Out of 12 tested measures, significant correlations to performance were found for conscientiousness (*r* = 0.30), emotional stability (*r* = 0.17), learning orientation (r = 0.32), problem-solving ability (*r* = 0.29), and error detection ability (*r* = 0.23). Other tested predictors, including extraversion and technical knowledge, did not have statistically significant relationships to superiors’ ratings. Strang (2020) tested if those working in cyber security were generally more suspicious than other military personnel. She found that cyber security personnel was more suspicious and that suspiciousness increased with the number of years of service in cyber security roles. Another study on a large database of job candidates found, that people in IT have lower work drive, are less open, less assertive, and more tough-minded (Lounsbury et al., 2014). Others have found that IT specialists of high proficiency are less agreeable than others (Witt and Burke, 2002).

Untested propositions based on analyses of cyber-related tasks or related literature have also been put forward. For instance, Dawson and Thomson (2018) suggested that the cyber workforce should consist of systematic thinkers, team players, people with a sense of civic duty, people willing to learn, good communicators, and people with both technical and social skills. An analysis of cyber-related occupations in the American occupational database O^*^Net found 19 abilities of relevance (Coovert et al., 2019). This analysis found that the general abilities of written comprehension, oral comprehension, oral expression, and written expression were ranked as important together with abilities, such as problem sensitivity (i.e., to detect anomalies or problems), deductive reasoning (i.e., to solve problems using general rules), inductive reasoning (i.e., to identify patterns), and information ordering (i.e., to arrange information according to a pattern). The same study used a panel of experts to rank the importance of these abilities and other abilities for roles in the US military. Attention to detail, analytical thinking, and information and technology attitude were ranked as most important; originality, mathematical reasoning, and emotional intelligence were ranked as least important.

Finally, predictors of success in the cyber security domain can be inferred from general relationships that are well established. For instance, motivation to learn (Colquitt et al., 2000; Gegenfurtner et al., 2016) is a known moderator of training effectiveness. Similarly, self-efficacy (i.e., self-ratings of competence) is a known predictor of performance (Stajkovic and Luthans, 1998).

## Cyber Test Future Soldiers

The establishment of a test battery for the Swedish Armed Forces included the development of several subtests to measure cyber knowledge and cognitive ability of particular relevance to cyber security. The test battery, *CTFS*, was designed to complement the existing generic enrolment test used in Sweden. The sections below provide a brief summary of the test along with the rationale for its design. The reader is referred to earlier publications for further details on the test (Lif et al., 2020).

### Background and Scope

In Sweden, potential conscripts are initially tested for interests and various physical abilities. They conduct a set of tests that measure general cognitive abilities and often they undergo interviews with a psychologist. Based on the results of the initial testing, further tests are conducted with selected subsets of the conscripts to identify people with a particular suitability for specific training, for example, to become a language interpreter or radio operator. *CTFS* was designed to be such a complementary follow-up test, aiming to improve the prediction of current enrolment tests regarding suitability for enrolment as cyber soldiers. One important component of the current enrolment tests is the tests for cognitive ability, named *I-prov 2000* (Carlstedt, 2002; Carlstedt and Gustafsson, 2005). *I-prov 2000* consists of ten sections: two non-verbal problem-solving sections, three verbal sections, four sections on spatial ability, and one section for technical understanding. Since *I-prov 2000* measures cognitive abilities, care should be taken to design the ability part of *CTFS* to complement those measurements, not repeat them. In addition to *I-prov 2000*, the following was considered in the design of *CTFS*:

- The content of other tests with similar aims (section “Related Work”)
- Generic analyses of cyber security work (in particular Newhouse et al., 2017)
- Task descriptions for future cyber soldiers
- Observations of subject matter experts during exercises
- Interviews with subject matter experts in the Swedish Armed Forces

The role of a cyber soldier is vaguely defined and the service will involve a range of tasks related to cyber defense, such as intrusion detection and vulnerability assessments. Thus, a test predicting performance on a number of tasks related to computers was needed. Since the role is new, the understanding of the cyber soldiers will improve over time and the role itself will most probably change as well. Therefore, the *CTFS* validity and reliability are assessed after each application and the *CTFS* components are up for revision in a continual refinement process. To date, this process has resulted in the *CFTS* test with multiple-choice questions that measure computer-related knowledge (six parts) and cognitive abilities believed to be of particular relevance to cyber security (four parts; Lif et al., 2020). The two subtests, on knowledge and ability, in *CTFS* are expected to measure different aspects, and results on these two subtest are not expected to have a strong correlation. The overall score on *CTFS* is obtained by summing scores on all parts. Confidentiality associated with the test and the role of future cyber soldiers prohibits descriptions of details about the items in the test. However, an overview of the parts together with their rationale is given below.

### Computer-Related Knowledge Subtest

The subtest for computer-related knowledge is included in *CTFS* for two reasons. First, the training of conscripts provided by the Swedish Armed Forces is fast-paced, and some initial knowledge in the field is believed to be necessary to succeed with the training. Second, existing knowledge is believed to be a good indirect measure of individuals’ motivation to learn things related to computers, and motivation is known to influence how effective learning interventions are (Colquitt et al., 2000; Gegenfurtner et al., 2016). Thus, it is expected that conscripts with a clear interest in computers will learn more during training and thus become better cyber soldiers.

The knowledge subtest in *CTFS* consists of six parts with multiple-choice questions with five alternatives and one correct answer. The parts comprise computers in general (e.g., binary numbers), computer security (e.g., encryption techniques), computer networks (e.g., network addresses), operating systems (e.g., memory management), programming (e.g., syntax), and web technology (e.g., html).

### Cognitive Ability Subtest

*I-prov 2000,* used by the Swedish Armed Forces contains ten sections meant to predict suitability for a wide range of roles in the military. The ability subtest in *CTFS* was designed to measure specific cognitive abilities believed to be of particular relevance for cyber security. It comprises one part that measures reasoning (e.g., rule induction), one part on mathematics (e.g., arithmetic), one part on error identification (e.g., spotting differences in code), and one part related to analytic ability (e.g., impulsivity).

As mentioned, the aim was that *CTFS* should complement the general cognitive ability parts in *I-prov 2000*. Therefore, the cognitive ability subtest in *CTFS* should not be regarded as a self-sufficient cognitive ability cyber test, but as an *additional* test following an initial screening on general cognitive ability. There are two main differences between the cognitive ability subtest and *I-prov 2000*. First, *CTFS* aims to measure other cognitive abilities. For instance, *I-prov 2000* does not have items specifically measuring error identification ability or the ability to induce rules. Second, the cognitive ability subtests are more difficult, and thus deliberately calibrated for individuals with higher cognitive ability compared to the *I-prov 2000* that aims at a much wider range of individuals.

## Materials and Methods

The Standard for Education and Psychological Testing (American Educational Research Association, American Psychological Association, National Council on Measurement in Education, 2014) was used to evaluate *CTFS*. This standard covers evaluation of validity and reliability, as well as whether the test is fair to the participants. This article focuses on the validity and reliability of *CTFS*, not its fairness.

### Participants and Procedure

The *CTFS* was taken by potential cyber soldier candidates as a part of the military mustering in 2020. This mustering process consisted of three phases: completion of a web-based form, general tests, and extended tests. The web form provides information on which candidates should conduct the conscription testing (*I-prov 2000*). The general tests include the cognitive ability test *I-prov 2000*, physiological tests, and psychological evaluations to select candidates for certain specialties, including cyber soldiers. Candidates for these specialties are subject to extended test procedures. For cyber soldiers, the extended test procedures comprised *CTFS* and a practical cyber test (not covered in this article).

During the extended test procedures, 62 candidates performed the *CTFS*. The test was taken using pen and paper over 3 h in a classroom setting. An instructor was present during the test to answer any questions that arose, and four observers were present to ensure that no person attempted to cheat. The candidates’ responses were collected on paper and converted to digital format using a document scanning software.

Of the 62 candidates who took the test, 30 were appointed to join the training to become conscript cyber soldiers. In this process, their *CTFS* results and the other parts of the extended test procedures were considered. In addition, all candidates who took the *CTFS* were excellent on prior testing, including the *I-prov 2000*. Thus, the 62 candidates who took the *CTFS* do not constitute a representative subset of all conscripts, and the 30 conscripts selected do not constitute a representative sample of the candidates who took the *CTFS*. The overall mustering procedure is presented in Table 1.

**Table 1:** Mustering procedures to select conscript cyber soldiers in 2020, and number of conscripts that conducted training.

Step	Information obtained/used
Web form sent to potential conscripts (N ≈ 100,000)	Health, physique, schooling, social life, personality, and attitudes
Candidates for general test procedures (N ≈ 15,000)	*I-prov 2000*, physical strength, physical endurance, visual ability, hearing ability, and psychological assessment
Extended test procedures for candidates (*N* = 62)	*CTFS* and practical cyber test
Cyber soldier training (*N* = 30)	–
Graduated cyber soldiers (*N* = 27)	Course scores and a second *CTFS* test

The candidates appointed to become cyber soldiers completed a number of courses as part of their training. Scores were collected from these courses. All scores were quantitative (points at course examinations) and were normalized to compute the average score for each person. However, it should be noted that the courses also involved assignments where conscripts collaborated, which may have distorted their individual scores somewhat.

As a step in assessing the reliability of *CTFS*, the cohort of 2020 took the test again before they graduated. The test content and procedure were the same as during the mustering.

### Sources Used for Validity Evidence

The authors used The Standard for Education and Psychological Testing’s view of validity as a unitary concept where validity evidence can be sought for from different sources (American Educational Research Association, American Psychological Association, National Council on Measurement in Education, 2014). Evidence from one such source alone would be insufficient for estimating the overall validity. Evidence for validity concerning internal structure, for example, does not necessarily mean that the test is valid, but it does constitute a prerequisite for a valid test.

#### Evidence Based on Test Content

The evidence for validity concerning test content came mainly from the development process. For the knowledge subtest of *CTFS*, approximately 100 items were initially produced by a team with expertise in cyber security, psychology, and statistics. These items were then screened and tested on participants similar to the target population as well as on cyber security experts. Item difficulty, discrimination, and distractors were analyzed and items were adjusted when needed. After these initial tests, a set of 36 items were selected for inclusion in the first version of the test.

For the cognitive ability subtest of *CTFS*, the selected parts were chosen based on interviews with subject matter experts, analysis of the general tasks of a cyber soldier, and analysis of related tests. Approximately 75 items were initially developed, primarily based on validated established tests for the chosen cognitive abilities. Of these items, 38 were selected after initial screening and tests that included analysis of item difficulty, discrimination, and distractors.

An analysis of item difficulty, discrimination, and distractors was also carried out on the results from the actual *CTFS* test taken by the conscripts, making the test development iterative for future selections.

#### Evidence Based on Internal Structure

The evidence for validity concerning internal structure was gathered by investigating the following correlations between candidates’ scores:

1. Items in relation to the subtest scores: All items in the knowledge and cognitive ability subtests are expected to have a positive correlation to their subtest total score.
2. Items in relation to the subtest parts: All items in each part of the knowledge and cognitive ability subtest are expected to correlate well with the overall score on their subtest part, more so than with the subtest as a whole.
3. The subtest parts in relation to each other and to the subtest total score: The knowledge subtest’s parts are expected to correlate with each other and to the overall knowledge subtest score. The cognitive ability subtest parts are expected to correlate in the same way.

These measurements, together with item difficulty analysis and item distractor analysis, are often described as being part of an *item analysis* (Kehoe, 1995; Rust et al., 2021). The item analysis thus aims at ensuring that items can discriminate between weak and strong test takers, that the items are not too easy or too hard, and that the item distractors (the wrong choices in a multiple-choice question) work as intended.

Both subtests and their individual parts were tested for normality using a Kolmogorov–Smirnov test. These tests showed that the normal distribution of the knowledge subtest and the ability subtest did not deviate from the estimated normal distribution (*p* > 0.05). However, as the participants’ results on some subtest parts contained data that were not normally distributed, non-parametric tests were chosen to examine how the different subtest parts correlated. Parametric tests were used only when all variables met the conditions for parametric data. This procedure was carried out to avoid overestimating the quality of the sample collected. For this reason, Spearman’s rank correlation, designated *ρ* (rho), was chosen (Field et al., 2012). In the analyses of each subtest part, point biserial correlation (rpb) was used, since one variable is binary.

#### Convergent and Discriminant Evidence

The *CTFS* ability subtest is expected to correlate with *I-prov 2000* since both of them measure cognitive abilities. However, the ability subtest was designed to complement *I-prov 2000* and the correlation is therefore expected to be moderate.

The cognitive ability subtest should not correlate highly with the knowledge subtest since they were designed to measure different variables. Similarly, the knowledge subtest is not expected to correlate highly with *I-prov 2000*.

#### Predictive Evidence

An initial analysis of the predictive validity of *CTFS* was conducted using the course scores as a measure of the degree of success for the cohort that completed the training. The predictive validity of *CTFS* was compared to the predictive validity of the more general *I-prov 2000* through bivariate and multivariate analyses.

As the selection of the 27 conscripts who finished training was performed in several stages using various criteria (Table 1), the sample was range restricted. That is, the 27 conscripts are not representative of the larger population. First, candidates with high scores on *I-prov 2000* (Stanine scale) were selected for extended test procedures. Second, candidates with high scores on *CTFS* were selected to become conscript cyber soldiers. Range restriction tends to attenuate a variable’s association to other variables. Thus, both the observed correlations between *I-prov 2000* and course scores and the observed correlation between *CTFS* and course scores will be lower in this dataset than what they would be if data on all potential candidates who took the web form (N ≈ 100,000) were included. Attempts were made to compensate for this effect.

As often is the case in analyses of this sort, numerous variables were involved in the selection procedure and it is not possible to obtain data on all selection variables’ relationships. The predictive validity of the *I-prov 2000* was compensated for using the method for direct (or explicit) range restriction (Dunbar and Linn, 1991):


$$R_{xy}=\frac{\left(\frac{S_{x}}{s_{x}}\right)r_{xy}}{\surd \left(1+\left(\frac{S_{x}^{2}}{s_{x}^{2}}-1\right)r_{xy}^{2}\right)}$$

Here, *S_x_* is the standard deviation for *I-prov 2000* in the unrestricted population with 100,000 potential conscripts; *s_x_* is the standard deviation in the restricted population comprising the 27 conscripts who completed training; *r_xy_* is the correlation between *I-prov 2000* and scores in the restricted population (the 27 conscripts); and *R_xy_* is an estimate of the true correlation between *I-prov 2000* and course scores in the unrestricted population (all 100,000 potential conscripts). Compensation for restriction of *CTFS* scores was made using the same method. However, as the true value for *S_x_* is unknown, three alternative hypothetical values are used. One with no range restriction at all (*S_x_/s_x_* = 1.00); one with the same restriction as in *I-prov 2000* (*S_x_/s_x_* = 2.25); one with the range restriction that was observed between the 62 candidates in the extended mustering procedures and the 27 conscripts who completed their training (*S_x_/s_x_* = 1.17).

To assess the incremental validity of the *CTFS*, both *CTFS* and *I-prov 2000* were used as predictors and the coefficient of multiple correlation was calculated. When the coefficient of multiple correlation between grades and the combination of *I-prov 2000* and *CTFS* is calculated, the correlation between the *I-prov 2000* and *CTFS* is of relevance. The observed correlation in the sample was used for this calculation. Compensation for range restriction was made using the same direct method as used elsewhere.

### Reliability

Internal consistency was analyzed using Cronbach’s Alpha for the knowledge and ability subtests of *CTFS* using data from all the 62 candidates who took the test.

Test–retest reliability of *CTFS* was calculated by comparing the *CTFS* results of the 27 conscripts from the testing during the mustering with a repetition of the test, taken after they completed their training. The correlation between the two scores was used as a measure of reliability.

## Results

The results are structured according to The Standard for Education and Psychological Testing (American Educational Research Association, American Psychological Association, National Council on Measurement in Education, 2014). As described earlier, validity evidence based on test content was mainly collected during the earlier phases of the development of *CTFS.* The sections below describe validity and reliability. Validity is described through evidence based on internal structure, and through convergent, discriminant, and predictive evidence. The evidence based on internal structure was primarily generated through an item analysis; convergent, discriminant, and predictive evidence were assessed by comparing the test score to other variables including a measure of degree of success for the candidates. Reliability was assessed through both internal consistency and a test–retest procedure.

### Validity: Evidence Based on Internal Structure

This section describes the results of the item analysis of the *CTFS*. The item analysis evaluated:

1. Items in relation to the subtest scores
2. Items in relation to the subtest parts
3. The subtest parts in relation to each other and to the subtest total score

First, items were analyzed in relation to their subtest. If a question showed a negative correlation, a correct answer to this question did not correspond with the total subtest score and the question should be revised. For the knowledge subtest (36 questions), one question had a negative correlation (−0.05) and six questions had non-significant correlations (*p* > 0.05) between 0.07 and 0.24. The remaining 29 questions had significant correlations (*p* < 0.05) between 0.26 and 0.57. For the ability subtest (38 questions), one question had a negative correlation (−0.05) and ten questions had non-significant correlations (*p* > 0.05) between 0.00 and 0.24. Moreover, two questions were too easy since all participants answered them correctly. The remaining 25 questions had significant correlations (*p* < 0.05) between 0.27 and 0.72. Overall, the item analysis for the knowledge and ability subtests suggested that items measure what they intend to do, with 69 of 74 questions having significant correlations with their respective subtest.

Second, item relations to their subtest parts were analyzed. The knowledge subtest showed that all questions had significant correlations (*p* < 0.05) to their respective part within the subtest. For example, all questions about network had a significant correlation with the total sum of the network questions. Low correlations can arise, for example, if a question is too difficult or poorly formulated so all respondents need to guess the right answer, or if the question is too easy and all respondents answer it correctly. For the ability subtest, item relations to their subtests parts were analyzed. The analysis showed that two questions were too easy since every participant answered them correctly, and three questions had non-significant correlations (*p* > 0.05) between 0.14 and 0.22. The remaining 33 questions had significant correlations (*p* < 0.05) between 0.26 and 0.86 with their subtest part. These correlations were consistently higher than the correlations with their subtest.

Third, the relationships between the subtest parts were analyzed. Tables 2, 3 report correlations between the subtest parts within the two subtests. As shown in the tables, all parts are positively correlated to each other except for the error identification part and most correlations are significant. Most importantly, all parts, except error identification, were significantly correlated to the total subtest score. Thus, most subtest parts relate to each other and to the total subtest score as desired. The particulars associated with the error identification part are discussed further in section “Future Work on CTFS.”

**Table 2:** Correlation between scores on different parts of the knowledge subtest, and between parts and total subtest score.

	Security	Network	OS	Programming	Web	Total subtest
Computer knowledge	0.17	0.15	0.16	0.32^*^	0.25	0.50^*^
Security	1.0	0.42^*^	0.45^*^	0.31^*^	0.42^*^	0.69^*^
Network		1.0	0.43^*^	0.29^*^	0.35^*^	0.66^*^
OS			1.0	0.33^*^	0.55^*^	0.72^*^
Programming				1.0	0.39^*^	0.64^*^
Web					1.0	0.77^*^

* *Statistically significant p < 0.05*.

**Table 3:** Correlation coefficients between scores on different parts of the cognitive ability subtest, and between parts and total subtest score.

	Mathematical ability	Error identification	Analytical ability	Total subtest
Reasoning	0.40^*^	0.22	0.37^*^	0.80^*^
Mathematical ability	1.0	−0.14	0.66^*^	0.81^*^
Error identification		1.0	0.06	0.17
Analytical ability			1.0	0.77^*^

* *Statistically significant p < 0.05*.

### Validity: Convergent and Discriminant Evidence

The *CTFS* ability subtest has a moderate positive correlation (*r* = 0.61) with *I-prov 2000,* indicating that they measure similar constructs. This result is expected, because they are both designed to measure (fluid) general intelligence. When compensating for range restriction, as described in the next section, the correlation strengthens (*r* = 0.80).

The *CTFS* ability subtest has a non-significant correlation (*r* = 0.18) with the knowledge subtest indicating that they measure different constructs. Similarly, the knowledge subtest has a non-significant correlation (*r* = 0.17) with *I-prov 2000*. Compensated for range restriction, the correlation is still low (*r* = 0.29) and insignificant. This result is also expected, because the knowledge subtest does not measure (fluid) general intelligence, but textbook knowledge of relevance to the cyber domain.

All values are presented in the Appendix.

### Validity: Predictive Evidence

The scores (normalized between 0 and 100) obtained by the conscripts during their training were used to evaluate the predictive validity of *CTFS*, or in other words, its usefulness in identifying recruits who are easy to train in cyber security. These data are available for the 27 cyber soldiers who completed their training.

Table 4 reports the predictive validity obtained when different variables are used to predict the cyber soldiers’ course scores. The second column in Table 4 reports observed validity values of models without compensation for range restrictions. As shown, *I-prov 2000* has a predictive validity of 0.22, and thus explains 5% of the variance in course scores. The validity of *CTFS* is significantly higher. The correlation coefficient of *CTFS* (overall) and scores is 0.52, corresponding to 27% explained variance. *CTFS* (overall) is the arithmetic mean of the scores of the two subtests. The subtest measuring cognitive ability has a stronger relationship to course scores (*r* = 0.61) than the subtest measuring knowledge (*r* = 0.12). The second column of Table 4 also reports the uncompensated predictive validity when *CTFS* is added to a linear prediction model together with *I-prov 2000*. The *CTFS* cognitive ability overlaps with *I-prov 2000*, with a positive correlation between the two predictors (*r* = 0.61).^1^ However, *CTFS* cognitive ability still increases the predictive validity considerably (Δ*R* = 0.42). The correlation between *I-prov 2000* and *CTFS* knowledge is low (*r* = 0.17), and the incremental validity of *CTFS* knowledge is inconsiderable (Δ*R* = 0.02). The correlation between *CTFS* overall and *I-prov 2000* is moderate (*r* = 0.51), and a substantial increase in validity (Δ*R* = 0.30) is obtained when *CTFS* is added.

**Table 4:** Validity of models predicting cyber soldiers’ course scores with observed data and compensation for range restrictions.

Prediction variables				
	Validity, observed	Validity, adjusted^a^	Validity, adjusted^b^	Validity, adjusted^c^
*I-prov 2000*	0.22	0.45	0.45	0.45
*CTFS* cognitive ability	0.61	0.61	0.67	0.87
*CTFS* computer knowledge	0.12	0.12	0.14	0.26
*CTFS* overall	0.52	0.52	0.58	0.81
*I-prov 2000* & *CTFS* cognitive ability	0.64	0.61	0.69	0.94
*I-prov 2000* & *CTFS* computer knowledge	0.24	0.45	0.45	0.47
*I-prov 2000* & *CTFS* overall	0.52	0.53	0.58	0.74

a *I-prov 2000–course scores compensated (S_x_/s_x_ = 2.25); I-prov 2000–other variables compensated (S_x_/s_x_ = 1.72); and CTFS–course scores uncompensated (S_x_/s_x_ = 1.00)*.

b *I-prov 2000–course scores compensated (S_x_/s_x_ = 2.25); I-prov 2000–other variables compensated (S_x_/s_x_ = 1.72); and CTFS–course scores compensated (S_x_/s_x_ = 1.17)*.

c *I-prov 2000–course scores compensated (S_x_/s_x_ = 2.25); I-prov 2000–other variables compensated (S_x_/s_x_ = 1.72); and CTFS–course scores compensated (S_x_/s_x_ = 2.25)*.

As mentioned in section “Predictive Evidence,” the observed values attenuate the incremental validity because the sample has been restricted to candidates with high scores on *I-prov 2000* and candidates with high scores on *CTFS*. The range restriction of *I-prov 2000* is known. The standard deviations of the entire conscript population and the 27 who completed the training are used to compensate for how the range restriction attenuate the relationship between *I-prov 2000* and course scores. The standard deviation for the entire conscript population and the 62 who took the *CTFS* are used to compensate for how the range restriction attenuates the relationship between *I-prov 2000* and *CTFS*. The range restriction of *CTFS*, however, is not known. Table 4 therefore reports the validity with different compensations for the range restriction of CTFS:

- *CTFS*–course scores uncompensated (*Sx/sx* = 1.00).
- *CTFS*–course scores compensated to the observed restriction that occurred when soldiers were selected for the extended mustering procedure (*S_x_/s_x_* = 1.17).
- *CTFS* compensated to the same extent as the *I-prov 2000* (*S_x_/s_x_* = 2.25).

As shown, compensation for range restriction increases predictive validity estimates considerably. Thus, in the hypothetical situation where all conscripts took the tests and were appointed to become cyber soldiers, the combined predictive validity is estimated to be between 0.53 and 0.74. *CTFS* provides incremental validity even if no compensation is made for how the range restriction influences *CTFS*’s relationship to course scores. Even with a conservative compensation, limited to the restriction that was observed since the extended mustering procedures, the incremental validity is considerable (Δ*R* = 0.13). Thus, *CTFS* complements *I-prov 2000* well.

### Reliability

Internal consistency was analyzed using Cronbach’s Alpha for the knowledge and ability subtests of *CTFS* using data from all the 62 candidates that took the test. The knowledge subtest had a reliability coefficient of 0.79 and the ability subtest 0.86.

Of the 30 conscripts selected to serve as cyber soldiers, 27 repeated the test just before they finished their training, approximately 15 months later. This retest allowed the authors to estimate the reliability of the different parts of the test and the reliability of the test as a whole. The correlation coefficients of this test–retest showed significant correlations (*p* < 0.05) for the knowledge subtest at 0.64, for the ability subtest at 0.57, and for the test overall at 0.60 (Table 5).

**Table 5:** Reliability for *CTFS* overall, the subtests and their parts.

Test-retest reliability	*r*
Overall test	0.60^*^
Knowledge subtest	0.64^*^
General computer knowledge	0.61^*^
Security	0.29
Network	0.35
Operating systems	0.22
Programming	0.21
Web	0.39^*^
Cognitive ability subtest	0.57^*^
Reasoning	0.40^*^
Mathematical	0.57^*^
Error identification	0.48^*^
Analytical ability	0.25

* *Statistically significant p < 0.05*.

The results from the internal consistency measures indicate that *CTFS* is stable when the test context is constant. The big difference between reliability from internal consistency and test–retest reliability is further deliberated in the discussion section.

## Discussion

The purpose of *CTFS* is to predict how well candidates will perform as cyber soldiers. This article has presented the test’s two components and an initial evaluation of its qualities. The sections below discuss the method used in the evaluation (section “Methodological Issues”), what the evaluation means in terms of validity (section “Validity of CTFS”), and future work related to the test (section “Future Work on CTFS”).

### Methodological Issues

*CTFS* was evaluated according to the principles of the Standard for Education and Psychological Testing (American Educational Research Association, American Psychological Association, National Council on Measurement in Education, 2014) as described earlier. Input from subject matter experts and traditional item analysis were used as a source of validity evidence based on test content and internal structure. Predictive validity evidence was collected using cyber soldiers’ course scores as a measure of degree of success. Reliability was evaluated using measures of internal consistency on the whole population that took the *CTFS* test, and test–retest reliability was evaluated on the sample that was selected and completed their training. The statistical practices used in the evaluation are well established. Nevertheless, there are limitations and issues associated with this evaluation.

First, the sample used in this research was small. Only 62 candidates took *CTFS*, data on course scores are only available for 27 conscripts, and only these 27 conscripts were retested. These are small samples, and all correlations between tests are not statistically significant. Furthermore, they do not represent random samples. Validity estimates should therefore be interpreted cautiously.

Second, observations were made on a set of candidates selected out of a large population. These candidates were selected especially to be suitable as cyber soldiers and are not representative for the population at large. One effect from this selection is that variables are restricted and validity estimates are attenuated. That is, *CTFS* and *I-prov 2000* would predict course scores better in the hypothetical situation if all conscripts had taken the test and obtained course scores. It is a fact that the *I-prov 2000* results were an important variable when candidates were selected for extended testing procedures, and this knowledge was used to estimate the true predictive validity based on a direct range restriction condition. However, interest, physical abilities, psychological attributes, and other variables are likely to have played a part. Thus, the estimates of how the range restriction attenuated relationships are based on a simplification.

Third, predictive validity was evaluated against course scores. The course score variable is a relevant measure of performance and success, but the aims of *CTFS* are broader and other outcome variables are conceivable. For instance, scores were obtained from academic courses known to load heavily on general intelligence, and the knowledge subtest may be a more useful predictor of peer-ratings, ratings by superiors, and performance in cyber security exercises. Unfortunately, none of these outcome measures are currently available.

### Validity of CTFS

Putting aside the uncertainty concerning the small sample size and the limitations of course scores as a success measure, *CTFS* appears to fulfil its aims. That is, it helps the Swedish Armed Forces to select candidates suitable as cyber soldiers. In the observed data, *I-prov 2000* predicted 5% of the variance in course scores while 27% of the variance is predicted if *CTFS* is used together with *I-prov 2000*. With a conservative estimate of how range restriction attenuates the predictive validity, *CTFS* increases the explained variance from 20 to 34%. There are few other similar tests where validity has been estimated. The *Cyber Test* is an exception (Trippe et al., 2014). When the *Cyber Test* was added to *Armed Forces Qualification Test*, validity increased by less than 1% (Trippe et al., 2014). Part of this difference may be due to the *Armed Forces Qualification Test* having a stronger relationship to course scores than *I-prov 2000* (*r* = 0.73 vs. *r* = 0.45), and it is unknown whether *CTFS* would complement the *Armed Forces Qualification Test* better than the *Cyber Test* does. However, *CTFS* adds a predictive power and successfully complements the general cognitive ability measured with *I-prov 2000*. Furthermore, *CTFS* can be used as a sole predictor of performance on cyber security courses, and explain somewhere between 27 and 66% of the variance in scores.

Internal consistency, a prerequisite for validity, was estimated using Cronbach’s alpha to be 0.79 and 0.86 for the knowledge subtest and ability subtest of *CTFS*, respectively. Thus, they fell within an acceptable range. The correlation between scores on *CTFS* before selection and after graduation, however, was 0.60. For knowledge tests, a reliability of at least 0.7 is generally considered good enough, and for ability tests 0.8 or higher (Rust et al., 2021). Schuerger and Witt (1989) found that reliability for intelligence tests correlated with the age of the participants, and the interval between tests. Tests on participants around 18–24 years old taken with 1 year in-between, and with test conditions similar to those in the present paper, have a reliability of around 0.85. Thus, in comparison to these tests, *CTFS* has a mediocre test–retest reliability. However, Schuerger and Witt (1989) also reported that small sample sizes are associated with lower reliability. As Table 5 shows, half of the subtest parts in the test–retest analysis had significant correlations in this small sample (*N* = 27). Thus, it is possible that higher reliability would be obtained with larger sample size. However, it is also possible, and likely, that a fair share of the candidates’ scores on *CTFS* are contingent on guessing answers and external factors, such as sleep before the test occasions. Nevertheless, the low test–retest reliability will be further investigated in the ongoing development of *CTFS*.

Another prerequisite for validity is the internal structure of the test. Almost all test items correlate with their subtest and subtest part, and most subtest parts correlate with their subtest as expected. There are a few weak parts of the test requiring further work and further validation. In particular, scores on the error identification part had weak insignificant correlations to the overall score on the cognitive ability subtest. This problem is partly due to poor calibration of the items’ difficulty, but it may also come from failure to capture the error detection ability.

When practical utility of *CTFS* is to be assessed, it is important to consider the intended use case for the test. *CTFS* is intended to be used as a complementary measure of cyber security aptitude or cyber security talent together with other measures. Tests of practical skills, psychological evaluations, and other measures may complement or overlap with *CTFS*. The utility of *CTFS* will depend on how it is used in a selection process. If practical tests have already been used to select candidates in an earlier stage, and these practical tests overlap with CTFS, the utility of *CTFS* would be lower than what is reported here. Conversely, if candidates have been selected based on their score on *CTFS*, the utility of adding practical tests to further select candidates will be lower than without *CTFS*. In this vein, the validity of *CTFS* demonstrated in this article is contingent on the relationship to *I-prov 2000*, which measures general cognitive abilities and is used earlier in the selection process. As intended and desired, the overall score on *CTFS* is moderately correlated to scores on *I-prov 2000*, but it is still important for performance in cyber security courses. The higher correlation between *I-prov 2000* and *CTFS ability* when adjusted for range restriction (*r* = 0.80) indicates that care should be taken in the ongoing development of this subtest so that the overlap between the two tests does not increase.

### Future Work on CTFS

*CTFS* is still under development. This article reports on its first real application as an instrument for screening candidates’ aptitude or talent in cyber security. As described above, the evaluation demonstrates that *CTFS* adds predictive power to the test previously available (*I-prov 2000*), but also that there is room for improvement. In the future, the aim is to (1) explore alternative operationalizations of the part on error identification and further refine the test, (2) run further *CTFS* tests on other cohorts, and 3) include other performance measures.

The part on error identification has a substantial theoretical support. Coovert et al. (2019) identified the ability of “problem sensitivity” (to detect anomalies or problems) as important in cyber security work and the domain experts interviewed during the development of *CTFS* stressed the importance of error detection ability in tasks, such as log file analysis (i.e., reviewing, interpreting, and understanding computer-generated records). In the current operationalization of the error identification subtest part, participants are presented with alternative pieces of information (e.g., text or code) and are asked to identify the deviating one. This operationalization was developed in the absence of suitable established test procedures for the ability. It is unclear if the weak results associated with this test part is because of a poor relationship between error identification ability and cyber security work, or if the current operationalization of error identification ability is poor. Existing error identification subtest part and new developed subtest part for error identification will be further evaluated to investigate the relationship between error identification (or problem sensitivity) and performance measures.

The analysis in this article used data obtained when *CTFS* was used in 2020 and course scores from the conscripts’ education. *CTFS* has since been used in 2021 and is planned to be used in 2022. In addition, a variant of the *CTFS* targeting selection for the cyber officer program in the Swedish Armed Forces is under development. Further evaluations will be possible as scores on these tests and associated performance measures become available.

From a research perspective, it would also be desirable to test *CTFS* against other performance measures than course scores. For instance, measures, such as peers’ ratings, superiors’ ratings of performance in exercises, and career advancement, may yield different results. Confidentiality associated with cyber soldiers may prohibit collection and analysis of such data for validation of *CTFS*. However, should data collection become possible, such analyses will be performed.

## Data Availability Statement

The datasets presented in this article are not readily available because of confidentiality. Since the tests will continue to be used for selecting conscripts, actual items cannot be shared publicly. Requests to access the datasets should be directed to patrik.lif@foi.se.

## Ethics Statement

Ethical review and approval was not required for the study on human participants in accordance with the local legislation and institutional requirements. The participants provided their written informed consent to participate in this study.

## Author Contributions

All authors have actively participated in the writing of this article. However, PL, TS, and P-AA have carried out a larger part of the work than CV and DE, in particular, the statistical analyzes. All authors contributed to the article and approved the submitted version.

## Funding

This work was supported by the Swedish Armed Forces.

## Conflict of Interest

The authors declare that the research was conducted in the absence of any commercial or financial relationships that could be construed as a potential conflict of interest.

## Publisher’s Note

All claims expressed in this article are solely those of the authors and do not necessarily represent those of their affiliated organizations, or those of the publisher, the editors and the reviewers. Any product that may be evaluated in this article, or claim that may be made by its manufacturer, is not guaranteed or endorsed by the publisher.

## Footnotes

^1^Correlations between the variables are provided in the Appendix.

## Appendix

Correlations between the tests are shown in below table.

**Table:**

	CTFS cognitive ability	CTFS computer knowledge	CTFS overall
*I-prov 2000*	0.61^*^ (0.80^*^)	0.17 (0.29)	0.51^*^ (0.71^*^)
*CTFS* cognitive ability		0.18	0.78^*^
*CTFS* computer knowledge			0.75^*^

*Correlations between CTFS subtests, CTFS overall, and I-prov 2000 based on the 62 candidates that took the CTFS test. For I-prov 2000, values in parenthesis show the adjusted values compensating for the range restriction occurring when only a small set of candidates who took the I-prov 2000 also took the CTFS. *Statistically significant p < 0.05.*
